# An integrative functional genomics approach reveals EGLN1 as a novel therapeutic target in KRAS mutated lung adenocarcinoma

**DOI:** 10.1186/s12943-021-01357-z

**Published:** 2021-04-06

**Authors:** Francesca Reggiani, Elisabetta Sauta, Federica Torricelli, Eleonora Zanetti, Elena Tagliavini, Giacomo Santandrea, Giulia Gobbi, Giovanna Damia, Riccardo Bellazzi, Davide Ambrosetti, Alessia Ciarrocchi, Valentina Sancisi

**Affiliations:** 1 Laboratory of Translational Research, Azienda USL-IRCCS di Reggio Emilia, via Risorgimento 80, 42123 Reggio Emilia, Italy; 2 Department of Electrical, Computer and Biomedical Engineering, University of Pavia, Pavia, Italy; 3 Pathology Unit, Azienda USL-IRCCS di Reggio Emilia, Reggio Emilia, Italy; 4 Clinical and Experimental Medicine PhD Program, University of Modena and Reggio Emilia, Modena, Italy; 5 Laboratory of Molecular Pharmacology, Department of Oncology, Istituto di Ricerche Farmacologiche Mario Negri IRCCS, Milan, Italy; 6 Department of Pharmacy and Biotechnology (FaBit), University of Bologna, Bologna, Italy

**Keywords:** Lung cancer, KRAS, CRISPR/Cas9 screening, EGLN1, HIF1A

## Main text

Despite extensive research and the recent introduction of innovative therapeutics, lung cancer remains the first cause of cancer-related death, with a 5 year survival of only 17% [1]. In lung adenocarcinoma (AD), the main lung cancer subtype, different driver genetic alterations can be targeted with specific small-molecule inhibitors [1], whereas KRAS mutations, which occur in about 30% of AD cases, have been traditionally considered undruggable. Current treatment approaches for KRAS-mutated patients include platinum-based chemotherapy or immune checkpoint inhibitors [1]. Multiple attempts have been done to develop molecules targeting RAS-mutated tumors, including GTP competitive inhibitors, farnesyltransferase inhibitors and compounds inhibiting downstream effectors, like MEK inhibitors or CDK4/6 inhibitors [2]. Recently, a new class of inhibitors has been developed, acting specifically on the KRAS G12C mutant and blocking it in the GDP-bound state [3]. These inhibitors are currently in clinical trials, showing promising early results, and may enter clinical practice in the next years [4]. However, more than half of lung cancer KRAS mutations are not actionable by these agents [3]. The lack of KRAS inhibitors clinically effective for all patients, together with the possible development of resistance mechanisms, emphasize the need of a deep molecular characterization of KRAS-driven AD, aimed to define new or overlooked targets.

In this work, we performed an integrative functional genomic analysis, combining in vitro dependency data within a large collection of cancer cell lines, gene druggability information and patients’ transcriptomics and mutational data. Through this approach, we identified and validated the EGLN1 gene as a novel druggable dependency, preferentially associated with KRAS-mutated lung AD.

### Identification of lung AD dependencies associated with KRAS mutation

To identify dependency genes that can be used as new therapeutic targets, we performed a CRISPR/Cas9 screening in the A549 cell line, derived from KRAS-mutated lung AD [5].

**Fig. 1:**
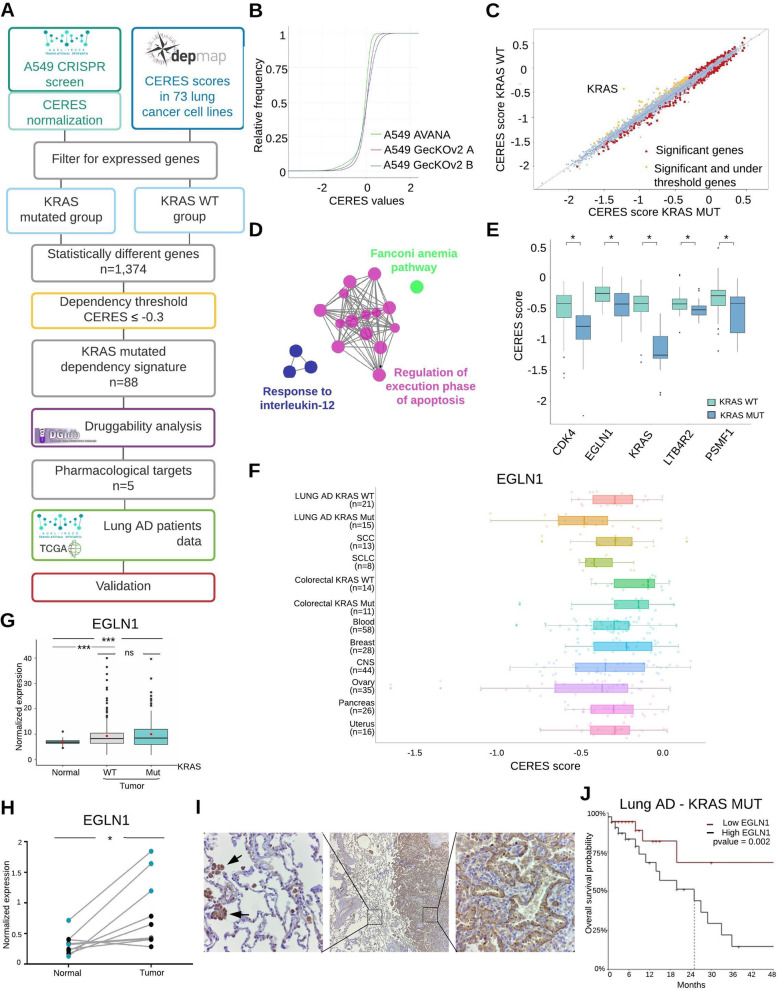
Identification of EGLN1 as a druggable pro-oncogenic factor in KRAS-driven lung AD. **a** Schematic representation of the bioinformatics and functional genomics integrative approach. **b** Cumulative distribution of CERES scores in A549 cells generated with AVANA library (DepMap data) or GeCKOv2 sub-libraries A or B (our screening data). **c** Comparison between median CERES scores in KRAS mutated (MUT) vs KRAS wild-type (WT) lung cancer cell lines. The genes (*n* = 1374) with a CERES score significantly different between the two groups (Wilcoxon test pvalue< 0.05) are highlighted in red. The genes (*n* = 88) with a CERES score significantly different between the two groups (Wilcoxon test pvalue< 0.05) and lower than our dependency threshold (*D* ≤ − 0.3.) are highlighted in yellow. **d** Network representation of significant enriched pathways for the 88 essential genes in the KRAS-mutated cell lines. **e** Comparison of CERES score distributions across the identified druggable targets in KRAS-mutated (MUT) vs KRAS-wild-type (WT) cell lines. **f** Comparison of CERES scores distributions for the EGLN1 gene in cell lines derived from different tumor tissues. Numbers between brackets on Y axis indicate the number of cell lines considered for each cancer type. **g** EGLN1 expression in TCGA lung adenocarcinoma patients cohort (*N* = 572, normal = 59, KRAS WT = 350, KRAS mutated = 154). **h** EGLN1 expression in a set of surgical samples from our Institute Biobank (*N* = 9). Cyan dots represent patients carrying a KRAS mutation. **i** Representative microscopy images of immunohistochemistry staining for EGLN1 expression in lung adenocarcinoma patients. Central image shows the interface between tumor and healthy lung tissue (50x magnification). Higher magnifications (400x) of the healthy lung epithelial tissue (left image) or of the tumor tissue (right image) are provided. Arrows indicate bright positive stained macrophages. The tissue has been counter-stained with hematoxylin. **j** Kaplan-Meier curve representing overall survival probability in KRAS-mutated patients presenting high (*N* = 39) or low (*N* = 39) levels of EGLN1 expression. *p < 0.05; **p < 0.01; ***p < 0.001; ns = not significant

To validate the results of our screening, we performed a data integration-based analysis of different and complementary -omics sources (Fig. 1a). First, we compared our screening data with essentiality data for 73 lung cancer cell lines, available through the DepMap portal [6]. DepMap data are normalized through the CERES algorithm, allowing to compare essentiality screening data of different cell lines and to assign a score to each gene, with the most negative scores assigned to the most essential genes [6]. We applied the CERES method to our screening data and, as shown by the cumulative distribution of CERES scores (Fig. 1b), our normalized results are consistent with DepMap data. Thus, we integrated our A549 dataset with the lung cancer DepMap data and used RNA-sequencing data to filter for expressed genes.

To identify dependency genes that were preferentially associated with the KRAS-mutated genetic background, we mined the dependency profiles of KRAS-mutated and KRAS wild-type lung cancer cell lines, extracting 1374 genes having a significantly different CERES score (Fig. 1c). To further refine this gene list, we selected genes showing a lower score in KRAS-mutated vs KRAS wild-type cell lines and we established a dependency (D) threshold on CERES scores at − 0.3, representing the mean value plus two standard deviations of CERES scores for common essential genes in A549 cells. By this step, we identified 88 genes, representing dependencies significantly associated with KRAS-mutated background. Enrichment analysis showed that this signature is significantly associated with apoptosis regulation and Fanconi anemia related pathways (Fig. 1d). Notably, both the apoptosis deregulation and DNA repair deficiencies are well-known alterations in KRAS-driven lung cancer, supporting the validity of our analyses [7, 8].

### EGLN1 as a novel druggable dependency

To select genes that can be targets of chemical compounds, we mined our signature querying the gene-drug interaction database [9]. In addition to KRAS, we identified four potential candidate genes (Fig. 1e), including the proteasome subunit PSMF1 and cycline kinase CDK4, thus confirming the already reported enhanced sensitivity to proteasome and CDK4 inhibitors in KRAS-mutated lung cancer [10, 11]. These results further support the validity of our approach.

Among these potential druggable candidates, we focused our attention on EGLN1, since its dependency has never been reported in KRAS-mutated lung AD. The EGLN1 gene encodes the PHD2 prolyl-hydroxylase, an oxygen sensor, regulating HIF transcription factor activity. Under aerobic conditions, EGLN1 hydroxylates the HIFα subunit, leading to the recognition by the VHL adaptor, which prompts the binding to ubiquitylation complexes and the consequent proteasome-mediated degradation. Conversely, in hypoxia, EGLN1 is inactive and the HIFα subunit is stabilized, activating the transcriptional programs that lead cells to hypoxia adaptation [12]. In line with the existence of multiple EGLN1-mediated processes, depending on the context, EGLN1 has been defined either as a tumor suppressor or an oncogene [13, 14].

We verified whether EGLN1 represents a shared dependency with other cancer types, comparing CERES scores in cancer cell lines of different origin (Fig. 1f). Notably, the cell lines most addicted to EGLN1 were the KRAS-mutated AD and ovarian cancer cells, confirming the known dependency on this gene in clear cell ovarian cancer [13]. Interestingly, in colorectal carcinoma, another KRAS-mutated neoplastic disease, EGLN1 scores were very close to zero in both KRAS-mutated and KRAS-WT cell lines, suggesting a specific dependency on this gene in lung cancer (Fig. 1f).

To gain insights into this novel role of EGLN1 in lung tumorigenesis, we evaluated its expression levels in both the TCGA adenocarcinoma cohort [15] and a set of patients’ samples retrieved from our Institute’s Biobank. Remarkably, EGLN1 expression, both at mRNA and protein level, was significantly higher in tumor tissue compared to surrounding healthy lung tissue (Fig. 1g-i). We also detected a strong EGLN1 expression in macrophages, indicating a possible role for this protein in tumor inflammatory microenvironment (Fig. 1i). Strikingly, high EGLN1 expression was also associated with a worse prognosis in the TCGA AD cohort and the difference in overall survival was even more pronounced in KRAS-mutated patients (Fig. 1j).

**Fig. 2:**
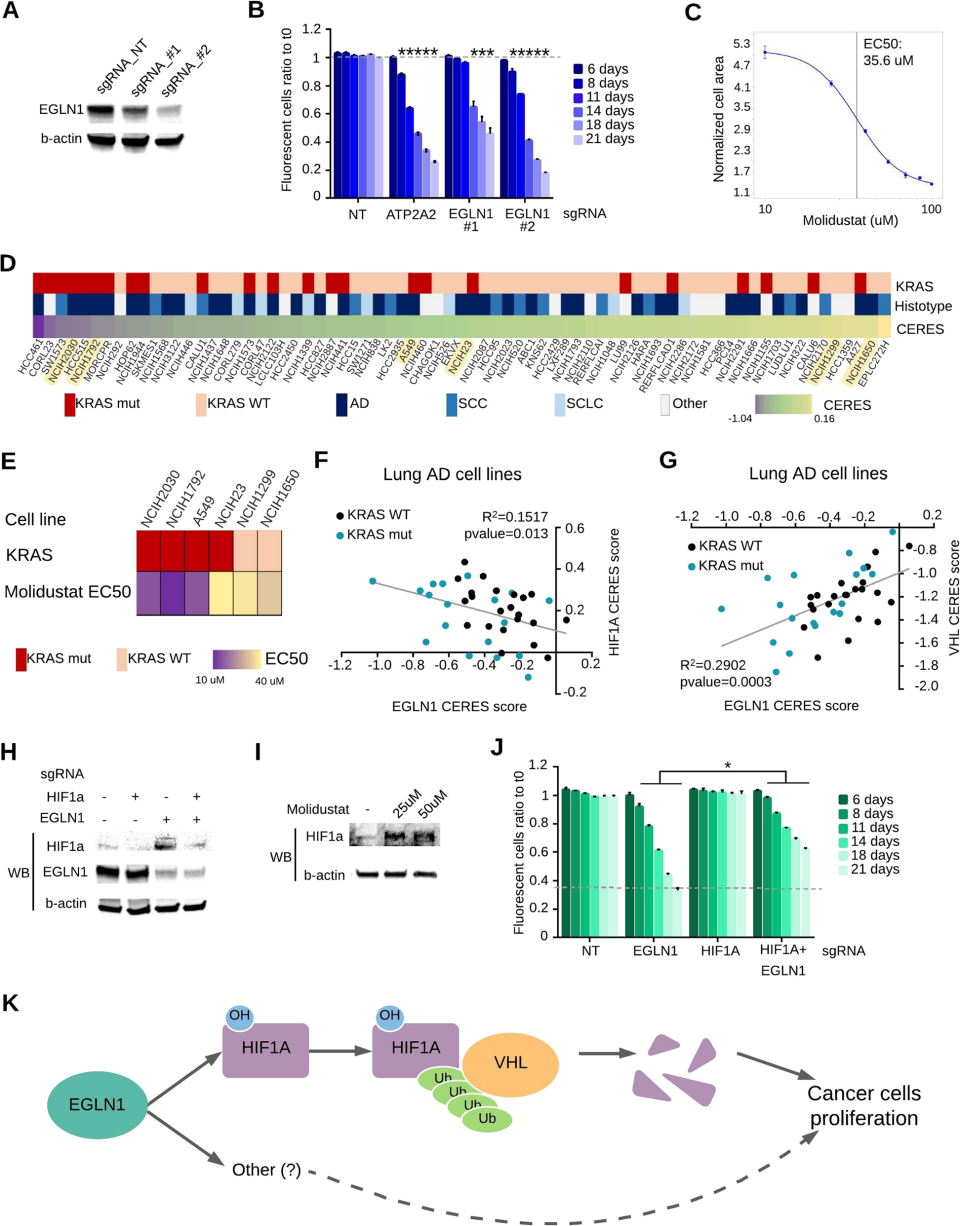
Dependency on EGLN1 is partially mediated by HIF1α stabilization. **a** Western blot analysis showing CRISPR/Cas9-mediated knockout (KO) of EGLN1 in NCI-H23 cells. A non-targeting sgRNA (NT) has been used as a negative control. β-actin is the loading control. **b** Competition assay showing reduced proliferation in NCI-H23 cells KO for EGLN1. Cells infected with a sgRNA targeting ATP2A2 gene are the positive control. For each time point the ratio between GFP-positive (infected) and GFP-negative (uninfected) cells has been calculated and normalized on T0. Statistical significance has been calculated comparing the normalized ratio for each sample with NT. Data are mean ± SEM; *p < 0.05; *N* = 3. **c** Sensitivity curve of NCI-H23 cells to EGLN1 inhibitor molidustat. Data are mean ± SEM; N = 3. **d** Heatmap showing lung cancer cell lines ordered by dependency on EGLN1 gene. **e** Heatmap showing sensitivity to EGLN1 inhibitor molidustat in a panel of lung cancer cell lines. **f** and **g** Correlation analysis between EGLN1 and HIF1A (**f**) or VHL (**g**) CERES scores in lung AD cell lines. Cyan dots represent KRAS-mutated cell lines, black dots represent KRAS-WT cell lines. **h** Western blot analysis for HIF1α or EGLN1 expression on NCI-H23/Cas9 cells infected with sgRNAs for HIF1A, EGLN1 or both. β-actin is the loading control. **i** Western blot analysis with anti-HIF1α antibodies, performed on NCI-H23 cells treated with molidustat for 72 h at the indicated concentrations. β-actin is used as loading control. **j** Competition assay performed on NCI-H23 cells. Cells infected with a non-targeting sgRNA (NT) are the negative control. NT, EGLN1 or HIF1A sgRNAs carried by a GFP-containing plasmid have been used to infect the cells. For double KO, EGLN1 sgRNA-GFP containing plasmid was used to infect cells which were already KO for HIF1A. For each time point the ratio between GFP-positive (infected) and GFP-negative (uninfected) cells has been calculated and normalized on T0. Statistical significance has been calculated comparing the normalized ratio for EGLN1 KO with double KO. Data are mean ± SEM; *p < 0.05; *N* = 3. **k** Schematic representation of the proposed mechanism

To validate the effect of EGLN1, we obtained the knock out (KO) in KRAS-mutated AD cell lines with two sgRNAs (Fig. 2a). As shown in Fig. 2b, the EGLN1 KO resulted in a significant proliferation impairment in a competition assay, with similar results obtained in different KRAS-mutated AD cell lines (data not shown).

Finally, to provide a proof of principle of the possibility to pharmacologically target EGLN1, we selected a panel of lung cancer cell lines and treated them with the EGLN1 inhibitor molidustat. All tested cell lines were sensitive to molidustat, with EC50 in the range of micromolar. Notably, KRAS-mutated cell lines displayed the highest sensitivity (Fig. 2c-e).

### EGLN1 pro-oncogenic activity is partially dependent on HIF1A

To gain further insights into the pro-oncogenic mechanism controlled by EGLN1, we relied on the notion that co-dependencies can be used to identify genes that have a similar function [16]. We found that EGLN1 CERES scores positively correlate with scores for the VHL gene and negatively correlate with HIF1A gene scores (Fig. 2f-g), suggesting that cancer vulnerability to EGLN1 inactivation may be related to its canonical function on HIF1α regulation. Indeed, EGLN1 KO or molidustat treatment induced HIF1α stabilization (Fig. 2h-i). To further investigate this hypothesis, we generated the double KO of HIF1A and EGLN1 genes (Fig. 2h). As shown in Fig. 2j, the HIF1A KO attenuated EGLN1 dependency, without completely rescuing the impaired cell proliferation observed in the single EGLN1 KO. Similar results were obtained with other KRAS-mutated AD cell lines (data not shown). These data indicate that, although HIF1α protein is stabilized by EGLN1 KO, its stabilization is only partially responsible for the detrimental effect on cell proliferation of the EGLN1 KO. We hypothesize that at least two mechanisms underlie the EGLN1 dependency in KRAS-mutated AD: one is HIF1α dependent, whereas the other is HIF1α-independent (Fig. 2k). This is partially contrasting with the data reported by Price and collaborators [13], showing that sensitivity to EGLN1 inhibitors requires intact HIF1α in ovarian cancer. Further investigation will be required to clarify additional mechanisms explaining EGLN1 dependency in KRAS-mutated lung AD.

## Conclusions

Overall, our results uncover a previously unknown pro-oncogenic function of the EGLN1 gene in KRAS-mutated lung AD. EGLN1 inhibitors are currently in clinical trials for anemia, demonstrating good tolerability and safety [17]. Our findings support the repurposing of these drugs to the lung cancer context, as single agent therapy or in combination with other compounds.

## Data Availability

The DepMap datasets for cell lines dependency screenings, RNA-sequencing, copy number and mutational profiles are available at https://depmap.org/portal/. Gene-drug interaction dataset is available at https://www.dgidb.org/. Mutational, RNA-sequencing and clinical data for TCGA cohort of lung AD patients is available at https://www.cancer.gov/tcga. All other datasets used and/or analyzed during the current study, together with materials and methods information, are available from the corresponding author on reasonable request.

## Abbreviations

AD: Adenocarcinoma
CRISPR/Cas9: Clustered regularly interspaced short palindromic repeats/ CRISPR associated 9
EGLN1: Egl-9 Family Hypoxia Inducible Factor 1
HIF1A: Hypoxia Inducible Factor 1 Subunit Alpha
KRAS: Kirsten Rat Sarcoma Viral Oncogene Homolog
NSCLC: Non-small cell lung cancer
TCGA: The Cancer Genome Atlas
VHL: Von Hippel-Lindau Tumor Suppressor
